# mTOR Links Tumor Immunity and Bone Metabolism: What are the Clinical Implications?

**DOI:** 10.3390/ijms20235841

**Published:** 2019-11-21

**Authors:** Azzurra Irelli, Maria Maddalena Sirufo, Teresa Scipioni, Francesca De Pietro, Amedeo Pancotti, Lia Ginaldi, Massimo De Martinis

**Affiliations:** 1 Medical Oncology Unit, Department of Oncology, AUSL 04 Teramo, 64100 Teramo, Italy; azzurra.irelli@hotmail.it (A.I.); teresa.scipioni@aslteramo.it (T.S.); panamedeo@libero.it (A.P.); 2 Department of Life, Health and Environmental Sciences, University of L’Aquila, 67100 L’Aquila, Italy; maddalena.sirufo@gmail.com (M.M.S.); fra722@hotmail.it (F.D.P.); lia.ginaldi@cc.univaq.it (L.G.); 3 Allergy and Clinical Immunology Unit, Center for the diagnosis and treatment of Osteoporosis, AUSL 04 Teramo, 64100 Teramo, Italy

**Keywords:** mTOR, tumor immunity, bone, metastasis, osteoimmunology, mTOR inhibitors, everolimus

## Abstract

Phosphoinositide 3-kinase (PI3K)/protein kinase B (AKT)/mammalian target of rapamycin (mTOR) plays a crucial role in the control of cellular growth, proliferation, survival, metabolism, angiogenesis, transcription, and translation. In most human cancers, alterations to this pathway are common and cause activation of other downstream signaling pathways linked with oncogenesis. The mTOR pathway modulates the interactions between the stroma and the tumor, thereby affecting both tumor immunity and angiogenesis. Inflammation is a hallmark of cancer, playing a central role in the tumor dynamics, and immune cells can exert antitumor functions or promote the growth of cancer cells. In this context, mTOR may regulate the activity of macrophages and T cells by regulating the expression of cytokines/chemokines, such as interleukin (IL)-10 and transforming growth factor (TGF-β), and/or membrane receptors, such as cytotoxic T-Lymphocyte protein 4 (CTLA-4) and Programmed Death 1 (PD-1). Furthermore, inhibitors of mammalian target of rapamycin are demonstrated to actively modulate osteoclastogenesis, exert antiapoptotic and pro-differentiative activities in osteoclasts, and reduce the number of lytic bone metastases, increasing bone mass in tumor-bearing mice. With regard to the many actions in which mTOR is involved, the aim of this review is to describe its role in the immune system and bone metabolism in an attempt to identify the best strategy for therapeutic opportunities in the metastatic phase of solid tumors.

## 1. Introduction

mTOR (mammalian target of rapamycin) regulates cell survival, proliferation, senescence, and metabolism [1]. mTOR is a multi-effector and ubiquitous protein, which is encoded by the *MTOR* gene [2]. mTOR is a protein that acts as a serine-threonine kinase and takes part in the formation of two complexes called mTORC1 and mTORC2. mTORC2 controls cell survival and cytoskeletal reorganization, while mTORC1 regulates protein synthesis and glucose utilization [3]. mTORC1 is stimulated during cellular activation so that T cell receptor (TCR) recruits PI3K (phosphoinositide 3-kinase) to the plasma membrane. The associated p110 subunit is then activated to phosphorylate phosphatidylinositol 4,5-bisphosphate (PIP_2_) and produces phosphatidylinositol (3,4,5)-trisphosphate (PIP_3_). PIP_3_ interacts with the pleckstrin homology domain of protein kinase B (AKT), causing a conformational change that allows PDK1 (kinase 3-phosphoinositide–dependent protein kinase-1) to partially activate AKT by phosphorylating threonine 308 (T308). Full activation of AKT is achieved by mTORC2-mediated phosphorylation at serine 473 (S473) and facilitates such processes as cell growth, cell cycle progression, and cell survival [4].

mTORC1 includes catalytic subunits of mTOR such as regulatory-associated protein of mTOR (RAPTOR), mammalian lethal with sec-13 protein 8 (MLST8), proline-rich Akt-substrate 40 kDa (PRAS40), and DEP domain-containing mTOR-interacting protein (DEPTOR). When mTORC1 is activated, it phosphorylates the effectors that are the main regulators of protein translation, including the regulation factors of ribosomal translation S6 kinase-1 (S6K-1) and 4E-binding protein 1 (4EBP-1) for the beginning of translation at the end of protein synthesis.

S6K-1 is the direct mTORC1 substrate that contributes to metabolic reprogramming by increasing glycolysis and protein, lipid, and nucleotide biosynthesis. mTORC1 also initiates powerful negative feedback regulation of growth factor receptor signaling, such that the inhibition of mTORC1 or S6K1 leads to elevated activation of PI3K, AKT, and the ERK pathway. S6K1 is highly sensitive to inhibition by rapamycin, and the disruption of S6K1-mediated negative feedback might contribute to the limited efficacy of rapamycin and rapalogs in cancer [5].

**Figure 1:**
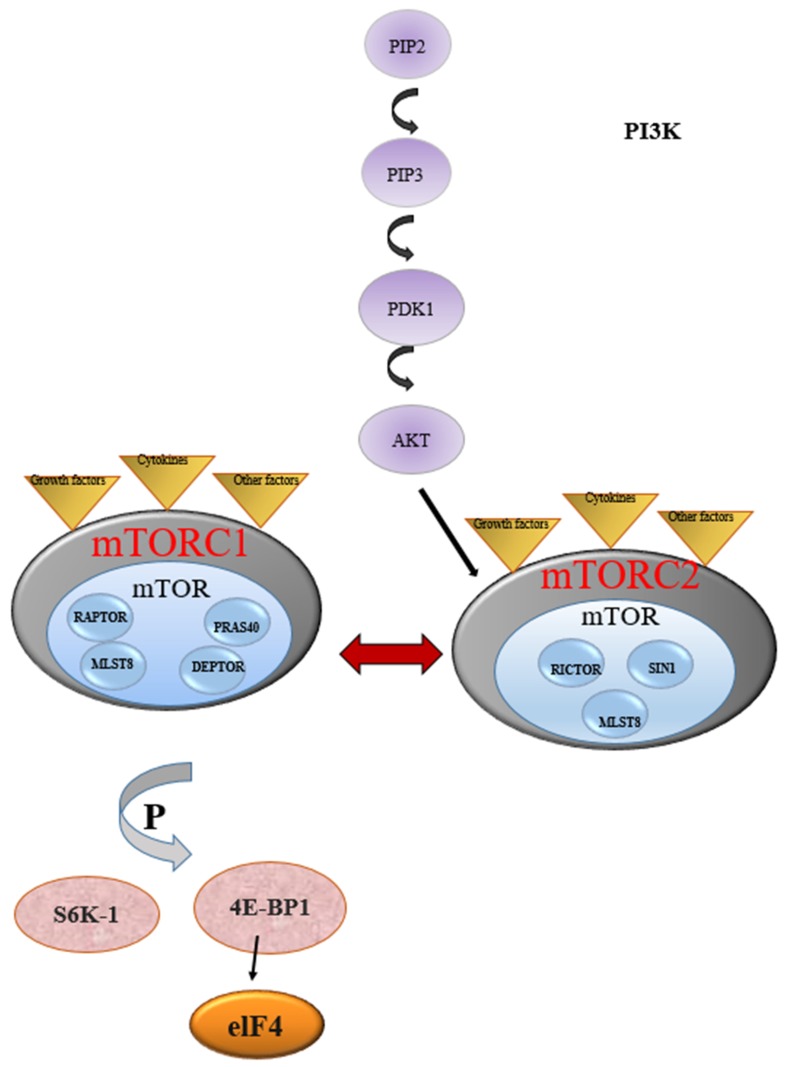
The phosphoinositide 3-kinase (PI3K)/protein kinase B (Akt)/mammalian target of rapamycin (mTOR) pathway. Activation of P13K phosphorylates phosphatidylinositol 4,5-biphosphate (PIP2) to form phosphatidylinositol-3,4,5-triphosphate (PIP3). PIP3 prompts the activation of downstream processes such as AKT, which transmits signals to effectors including mTOR complexes to enhance cellular processes. mTORC1 is stimulated during cell activation, whereby T-cell receptor (TCR) stimulates the activation of P13K. mTORC1 includes catalytic subunits of mTOR such as regulatory-associated protein of mTOR (RAPTOR), mammalian lethal with sec-13 protein 8 (MLST8), proline-rich Akt-substrate 40 kDa (PRAS40), and DEP domain-containing mTOR-interacting protein (DEPTOR). mTORC2 comprises three proteins: RICTOR, MLST8, and SIN1. Activation of mTORC2 occurs through the phosphorylation of AKT, while mTORC1, when activated, phosphorylates effectors that are major regulators of protein translation including translation-regulating factors ribosomal S6 kinase-1 (S6K-1) and eukaryote translation initiation factor 4E binding protein-1 (4EBP1) to enhance protein synthesis.

mTORC2, on the other hand, can be activated directly from PI3K and can phosphorylate and activate AKT and other related kinases. Furthermore, through the PI3K-AKT signal, the cytokine and TCR co-stimulatory signals can activate the mTOR signaling pathway to activate mTORC2 to follow T cells. mTORC2 comprises three proteins: RICTOR, MLST8, and SIN1. Activation occurs through AKT phosphorylation at serine-473 [6]. mTOR is activated by a series of upstream signaling pathways such as PI3K/AKT, RAS/MAPK/RSK and various growth factors and cytokines [7]. As mentioned above, AKT and mTOR are activated through the conversion of phosphatidylinositol-4, 5-bisphosphate (PIP2) to phosphatidylinositol-3,4,5-triphosphate (PIP3) in the cell membrane, from which a protein phosphorylation cascade is induced. This pathway (Figure 1) is the target of anticancer therapies [6]; being the deregulation of mTOR activity, it is associated with numerous types of cancer. The activity of mTORC1 is stimulated by growth factors, insulin and amino acids (in particular leucine), energy status, and oxidative stress. Insulin receptor substrate (IRS) activates PI3K through the stimulation of growth factors. PI3K generates phosphatidylinositol 3,4,5-triphosphate (PIP3) after phosphorylation. PIP3 therefore promotes phosphorylation of protein kinase (PKB/AKT) by 3-phosphoinositide-dependent protein kinase-1 (PDK1) [2]. Activation of mTORC1 causes phosphorylation of ribosomal protein S6 kinase 1 (p70S6K1 or S6K1) and 4E-binding protein 1 (4E-BP1), activating the starting factor of eukaryotic translation 4E (eIF4E). This cascade of events is negatively regulated by tuberous sclerosis TSC1-TSC2 complex, with inhibitory effects on 4E-BP1 and eIF4E [8].

mTORC1 is activated at the lysosome, in response to a variety of environmental signals, through two Ras-related small G proteins, Rheb- and Rag-GTPase. Multiple regulators have been identified to regulate the activation of Rag-GTPases, such as the Ragulator complex, GATOR1/2, CASTOR1, and Sestrin2. The Ragulator complex functions as the GEF (Nucleotide exchange factor) for RagA, while GATOR1 is identified as a GAP (GTPase-activating protein) for Rag. Senstrin2 mediates mTORC1 activity by acting as a GDI (guanine nucleotide dissociation inhibitor) for Rag or a protein partner with GATOR2. In contrast to Rag GTPase, the regulation mechanism of Rheb is less understood. The TSC complex, consisting of three core subunits (TSC1, TSC2, and TBC1D7), is identified as a major upstream regulator of Rheb. This complex negatively regulates mTORC1 activity by converting Rheb from its active form (GTP-bound Rheb) to its inactive form (GDP-bound Rheb). The GAP activity of TSC2 on Rheb is regulated by extracellular signals through the phosphorylation of TSC2 by AKT, AMPK, GSK3, ERK, SGK, or RSK. However, whether the TSC complex has any other function rather than as a GAP of Rheb is still unclear [9].

The activity of the so-called “tumor suppressor” TP73/p73 converges on TSC1-TSC2. TP73/p73 deficiency, by activating the autophagy mechanism involving AMPK-TSC-MTOR signaling, promotes autophagy of cancer stem cells (CSLCs) [10]. CSLCs reside in tumors, which mostly contain differentiated cells. Due to their self-renewal abilities, CSLCs remain refractory to various therapies that kill differentiated cancer cells; therefore, CSLCs are the main cells responsible for the failure of antitumor treatment. Autophagy induced by TP73/p73 deficiency occurs due to the reduction of ATP levels resulting from metabolic perturbations. The reduction of ATP levels leads to the activation of phosphorylated-AMP-activated protein kinase (p-AMPK), which activates the TSC1-TSC2 complex by phosphorylation. This complex inhibits the phosphorylation of mTOR, leading to the activation of autophagy [10]. 4E-BP1 and eIF4E are the main regulators of protein translation, cell proliferation, angiogenesis, and autophagy. Autophagy is an evolutionarily conserved system of self-degradation of cellular components through an autophagosomal-lysosomal pathway dependent on nutritional conditions and is reported to be deregulated in cancer. During hunger, mTORC1 is inactivated and dissociates from the ULK1 kinase complex (a protein complex composed of Atg1, Atg13, Atg17, and Atg101) to induce autophagy [2]. Dysregulated autophagy is involved in several pathological processes, including cancer; an increasing number of clinical anticancer trials are targeting the mTORC1-autophagy pathway, and inhibitors of autophagy might have beneficial effects on osteoporosis [11,12].

## 2. Bone and the Immune System

A close relationship exists between bone and the immune system. Bone is the key element in the skeleton supporting locomotricity in vertebrates; at the same time, bone has a crucial role in maintaining homeostasis of mineral metabolism and is the temple of the hemopoietic and immune systems. Cells involved in bone metabolism and hematopoietic cells engaged in immune responses derive from bone marrow precursors and cooperatively interact to carry out their specific functional activities while sharing the same microenvironment, influenced by similar mediators. One of the most relevant molecules linking bone and the immune system is the receptor activator of nuclear factor-kB (NF-kB) ligand (RANKL), which, to date, has been shown to serve multiple functions in both systems. Furthermore, a close relationship exists between immune regulation of bone turnover and tumor growth; the bone marrow microenvironment can become a niche for the development and progression of cancer [13].

RANKL is expressed on several cell types in proneoplastic inflammation and functions as a chemotactic factor that favors bone metastasis, while cancer cells often express RANK. Sometimes, cancer-associated inflammation may result in an unsuccessful opposition to tumor growth, and some patterns of immune activation could facilitate the development of tumors. Cancer cells in the skeleton activate osteoclasts that mediate bone resorption and supplemental release from the bone matrix of growth factors, resulting in tumor proliferation and impaired bone turnover, while osteoblasts in bone metastasis decrease osteoprotegerin production, inducing further osteolysis. RANK/RANKL is also essential for the proliferation of mammary epithelial cells. RANKL stimulates mammary stem cells involved in the oncogenic activity of estrogen and progesterone, and mammary-specific RANK overexpression ultimately results in an increase in tumors after treatment with progestin and carcinogens. The RANK/RANKL system was shown to have a role in breast cancer onset, while tumor-infiltrating regulatory T cells (Treg) are critical cells for maintaining RANKL expression in mammary tumors. Blocking RANK is a strategy capable of inhibiting bone resorption, decreasing tumor growth, enhancing apoptosis of malignant cells, and reducing proneoplastic inflammation. Macrophages have a role in tumor progression and in bone, and they support cancers such as breast and prostate, which preferentially metastasize to the skeleton. Tumor cells, macrophages, and skeletal cells orchestrate complex immune reactions. Osteal macrophages expressing CD68, a phagocytic capacity marker of cells infiltrating metastatic lesions, could facilitate tumor establishment and growth. Tumor-derived parathyroid hormone-related peptide (PTHrP) drives myeloid cell recruitment *via* osteoblast-produced chemokine (C-C motif) ligand 2 (CCL2), which is high in the bone microenvironment and whose levels are associated with poor prognoses in primary breast tumors [14].

mTOR signaling interacts with several molecular signaling pathways such as PPARy signaling, the canonical Wnt/b-catenin signaling pathway and the RANK/RANKL signaling pathway, thus participating in maintaining bone homeostasis. It is involved in the metabolic regulation, function, and differentiation of adaptive/innate immune cells and, given its key role in regulating fundamental biological processes, it is easy to imagine the tight connection with cancer onset and progression. 

## 3. mTOR and the Immune System

**Figure 2:**
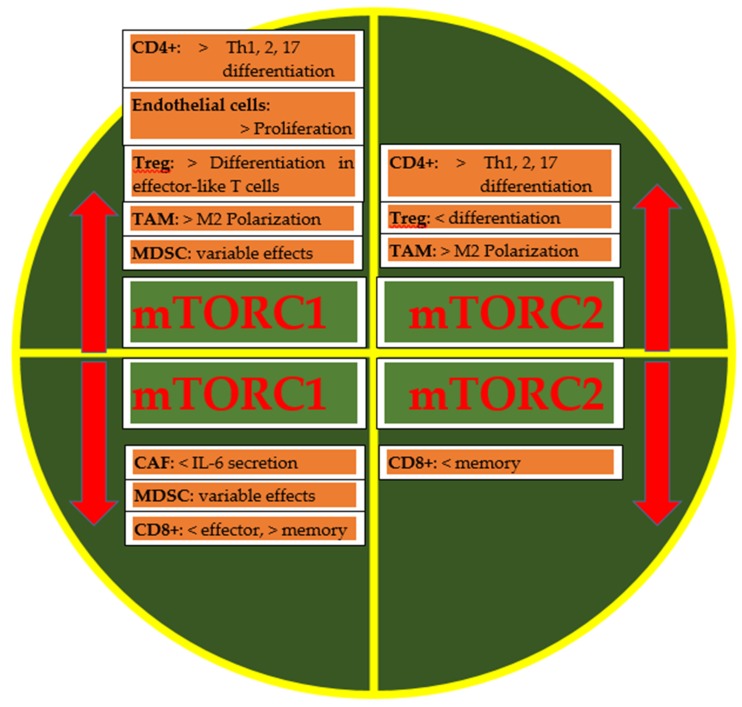
Immunoregulatory functions of mammalian target of rapamycin (mTOR) complexes in tumor microenvironment elements. An increase (red up-arrow) in mTORC1 activity induces Th1, Th2, and Th17 differentiation, endothelial proliferation, tumor-infiltrating regulatory T cells (Treg) differentiation in effector T cells, and M2 polarization; mTORC2 increased activity increases Th1, Th2, Th17 and M2 polarization and diminishes Treg differentiation; reduction (red down-arrow) of mTORC1 activity diminishes interleukin (IL)-6 production and CD8+ effector while increasing CD8+ memory; on the contrary, CD8+ memory is reduced if mTORC2 activity is diminished. mTOR complex 1: mTORC1. mTOR complex 2: mTORC2. Tumor-associated macrophages: TAM. Myeloid-derived suppressor cells: MDSCs. Cancer associated-fibroblasts: CAFs. < indicates a decrease in activity; > indicates an increase in activity.

The inhibition of mTOR increases immunosurveillance [15,16], modulating the interactions between the tumor microenvironment and tumor cells [17]. The tumor microenvironment includes immune system cells related to innate immunity (macrophages, natural killer cells, neutrophils) and acquired immunity (T helper lymphocytes, cytotoxic T lymphocytes, regulatory T lymphocytes). The macrophage engulfs the cancer cell and, through lysosomal enzymes, it digests and transforms its protein components into peptides, which are presented on the surface of the macrophage cell in the pocket of a class II molecule called major histocompatibility complex (MHC). This class II MHC complex and antigen is presented to T helper lymphocytes. The interaction between the macrophage and T helper lymphocyte is regulated by co-stimulatory and co-inhibitory molecules. When the T helper lymphocyte recognizes the antigen, it produces cytokines such as interleukin-2 (IL-2) that stimulate B lymphocytes to produce antibodies (humoral immunity) and stimulate cytotoxic T lymphocytes to kill cancer cells. Natural killer cells kill cancer cells through antibody-dependent cellular cytotoxicity (ADCC) and the imbalance between activating receptors and inhibitory receptors. In fact, when activating receptors prevail over inhibitory receptors, there is destruction by osmotic lysis of cells by natural killer cells. The programmed death-ligand 1 (PD-L1) is an immunomodulator expressed as a variable percentage among the various tumors, as well as on stromal cells of the microenvironment. The (PD-L1)/ Programmed death 1 (PD-1) interaction (present on the cytotoxic T lymphocyte) is inhibitory: the cytotoxic T lymphocyte is not activated. The cytotoxic T lymphocyte is able to produce IFN-gamma, which acts on an IFN-gamma receptor present on the tumor cell, increasing the expression of PD-L1 and PD-1. Thus, the tumor cell itself induces immunosuppression. Another co-inhibitory molecule targeted in tumor immunotherapy, besides PD-1, is cytotoxic T-Lymphocyte protein 4 (CTLA-4) [18,19,20]. mTOR is the regulator of cytotoxic T lymphocyte differentiation. A blockade of mTOR inhibits the proliferation of regulatory T lymphocytes with minimal effect on T helper lymphocytes and cytotoxic T lymphocytes. Furthermore, mTOR modulates the role of PD-L1 [17]. The inhibition of mTOR activity causes defective natural killer cell maturation, according to molecular mechanisms still unknown [21]. Several immunomodulatory functions of mTOR are shown in Figure 2.

mTOR regulates many immune cellular functions such as differentiation, activation of T cells, tumor-associated macrophages (TAM), and antigen-presenting cells. mTOR regulates memory CD8+ T cells’ differentiation, with different roles of mTORC1 that positively influence CD8+ T cells’ effector responses, and mTORC2 is involved in CD8+ T cells’ memory upregulation. CD4+ T cell differentiation is significantly affected by mTORC1 and mTORC2 activity, whose inhibition affects Th1, Th2, and Th17 differentiation. If the role of mTORC1 appears to be better characterized, that of mTORC2 seems to be more complicated and less defined. Actions of mTORC1 are reported on the differentiation of Th1, Th2, and Th17 [22]. Instead, the role of mTORC2 in T helper cell differentiation and effector function is more complex, with no obvious preference for Th1, Th2, and Th17 [22]. Naïve CD4+ T cells only differentiate into Treg in the absence of mTORC1 and mTORC2 activities [22]. mTOR mediates different Treg functions: an increase in mTORC1 activity increases differentiation in effector-like T cells, while an increase in mTORC2 activity diminishes differentiation. In the tumor microenvironment, there is a high number of tumor-associated macrophages, derived from tissue-resident macrophages or recruited by cytokines and chemokines, and classified on the basis of their polarization in M1 and M2. They share the same functions and phenotype [17], but to simplify, M1 has a role in antitumor response and phagocyte-dependent inflammation, while M2 is more tolerant towards tumor growth, inhibiting phagocytic function. They also differ in their chemokine and cytokine production with subsequent differences in antitumor activities (progression, angiogenesis). mTORC1 downregulation reduces inflammation and imbalances in macrophages’ M1 polarization. Inhibition of mTORC2 reduces the differentiation of M2 macrophages. M2 surface markers are upregulated by mTORC2 with increased invasion and metastasis in breast tumor models. The mTOR axis regulates the release of soluble factors involved in the recruitment of myeloid derived suppressor cells (MDSCs), a heterogeneous group of immature myeloid cells at various stages of differentiation, including precursors of macrophages, granulocytes and dendritic cells. However, the role of mTOR in MDSCs’ function is still controversial. To complete the tumor microenvironment (TME), there are the endothelium and fibroblasts, which represent two other fundamental components in the defense mechanisms or progression of the tumor, whose activity is regulated by different humoral factors and the interaction with the other cells of the microenvironment. Increase in mTORC1 activity favors endothelial cells proliferation, while its reduction diminishes IL-6 production by cancer-associated fibroblasts (CAFs), thus reducing their pro-tumorigenic properties. Although certainties are still lacking, several studies have shown that a certain connection exists between the proliferation of Treg and the transient inhibition of mTOR signaling. The level of nutrients and energy in cells is carefully monitored by mTORC1 and AMPK, and in cancer these signaling nodes are dysregulated. AMPK can be considered as an antagonist of mTOR activity that promotes aerobic glycolysis and anabolic metabolism; during tumorigenesis, AMPK is suppressed and mTORC1 is activated, whereas activation of AMPK is critical for energy balance and metabolic fitness of T effectors and memory cells. AMPK deficiency during T cell activation should enhance effector T cell development. There is evidence for reciprocal regulation of AMPK and mTOR involving phospholipase D (PLD) and its metabolite in cancer cells, and it has therapeutic implications [23]. Metabolic communication among cancer, T cells, and innate immune cells might contribute to modulating antitumor immune response and to the progression of cancer.

AMPK can inhibit glycolysis, anabolic metabolism, and T cell differentiation, and regulates the balance between Th17 and Treg. Metformin is an activator of AMPK able to inhibit Th1 and Th17 differentiation, to reduce inflammation in some mouse models and to increase Treg in some others. Cancer and T cells compete for energy and nutrients, and the abnormal metabolism of tumor cells inhibits the immune metabolism of T cells. A conversion of effector T cells into ineffective cells is the result of a weakened T cell glycolytic pathway and of a reduced capability of secreting cytokines by T cells [24].

## 4. mTOR and Bone Metabolism

Several factors, such as parathyroid hormone (PTH), can induce the expression of RANKL on osteoblasts in bone. The soluble sRANKL form acts on a receptor present on the surface of the monocytic precursor of osteoclasts (RANK), transforming them into activated osteoclasts. Osteoblasts produce osteoprotegerin (OPG), which binds RANKL by inhibiting the activation of osteoclast precursors [14,25,26]. Tumor cells can activate osteoclasts by stimulating the expression of RANKL by osteoblasts or by exhibiting a pseudo-phenotype similar to osteoblasts. Cathepsin G produced by tumor cells cuts the extracellular domain of RANK, generating sRANKL, which activates osteoclast precursors. The increase in bone turnover, also favored by a low dietary intake of calcium and vitamin D deficiency, promotes the growth of the tumor in the bone. During the metastatic process, the components deriving from bone metabolism, generally identified as formation markers (e.g., PINP and PICP) and bone resorption (e.g., NTx and CTx), are released into the bloodstream [27]. PTH induces osteoblasts to secrete macrophage colony-stimulating factor (M-CSF) and RANKL; both induce monocytes to differentiate into osteoclasts. M-CSF activates AKT, while RANKL activates NF-kappaB, converging on mTOR. Osteoblasts secrete osteoprotegerin (OPG) in order to counter RANKL. OPG binds RANKL to prevent its binding to RANK on monocytes [28,29]. Due to the OPG sequestration by tumor cells, a microenvironment is created that facilitates the expansion of cancer cells. Furthermore, OPG binds the TNF-α–related apoptosis-inducing ligand (TRAIL), a natural pro-apoptotic and antitumor factor. It follows that there is interest in combining anti-RANKL therapy with immune therapy [30]. RANKL expression is increased by 1,25-VD, PTHrP, IL-1, IL-6, TNF, prolactin, TNF, corticosteroids, and prostaglandin E2. RANKL/RANK is decreased by estrogens, calcitonin, transforming growth factor beta (TGF-β), PDGF, and OPG, which means the prevention of excessive bone resorption [31,32,33]. RANKL binds RANK, activating TRAF6 and SRC, followed by the activation of PI3K/AKT, NF-kB, and MAPK [28]. RICTOR is a subunit of the mTORC2 complex, which activates Akt/PKB signaling. Deletion of RICTOR in mature osteoblasts induces a significant reduction in bone turnover. This leads to an improvement in bone mass [34]. MTOR signaling in osteoblasts may not be essential for maintaining short-term bone homeostasis. The WNT7B gene induces a drastic increase in bone mass in osteoblasts due to the increase in the number of osteoblasts, significantly stimulated activity, and bone formation, but not when Raptor is absent. Therefore, Wnt-7B requires mTOR to promote bone formation. It is not known whether the mTOR pathway is important for osteocytes, but it is obvious that this pathway influences the biology of osteocytes. When oxygen and nutrients are not abundant, for example, in osteocytes that are not in direct contact with blood vessels, these cells slow down their metabolism and recycle cellular components through autophagy. Since the activation of mTOR improves the protein conversion process by consuming energy and inhibiting autophagy, it could be inferred that mTOR activity must remain low in unstimulated osteocytes. The alterations in mTOR activity could therefore have effects on osteocyte survival and bone metabolism. Exercise, which stimulates the production of IL-6 and IGF-1 in bone, can improve bone mass [35]. IGF-1 increases bone formation because it stimulates the osteogenic differentiation of bone precursors that form osteoblasts through the PI3K/AKT/mTOR pathway [36].

Tumor cells and osteogenic cells form heterotypic adherent junctions, which improve mTOR activity and guide bone colonization. VCAM-1 derived from tumor cells has been shown to bind osteoclast progenitor cells, inducing differentiation, and this cross-talk represents a critical step in the progression of microscopic bone metastasis to clinically significant bone metastasis. The tumor cells disseminated in the bone marrow establish their first foothold in the bone marrow, competing with hematopoietic stem cells for occupation of the pre-metastatic niche. The interaction between the osteogenic cell and the tumor cell leads to the activation of mTOR in the tumor cell. Therefore, the hyperactivation of mTOR leads to an increase in bone resorption but also to the transformation of the preneoplastic niche into micrometastases and then into metastasis [37].

## 5. Vitamin D: Between mTOR and the Immune System

Vitamin D (VD) synthesis starts from the action of ultraviolet light in the skin. Cholecalciferol is hydroxylated in 25-hydroxy-vitamin D (25-VD) in the liver via cytochrome P-450. In the kidney, calcitriol (1,25-VD, the active form) is synthesized via CYP27B1 and transported into the blood via the vitamin D-binding protein (VDBP). 25-VD inactivation in calcitroic acid (24,25-VD) is performed by CYP24A1 [38]. 1,25-VD stimulates the expression of DNA-damage-inducible transcript 4 protein (DDIT4), also known as a regulator in the development and response to DNA damage 1 (REDD1), which acts as a negative regulator of mTOR [39,40]. The combination of vitamin D and mTOR inhibitors inhibits osteoclast differentiation and their function in inflammatory conditions by increasing NFATc1 and decreasing cathepsin K expression [41]. VD inhibits T helper lymphocytes and upregulates PDL-1 expression on both tumor and immune cells [42]. VDR binds to 1.25-VD and dimerizes with RXR. The VDR/RXR complex translocates to the nucleus and induces the transcription of PLC-γ1, which catalyzes the hydrolysis of PIP2 into IP3 and DAG. IP3 increases cytoplasmic calcium levels, resulting in a nuclear import of NFAT-1. DAG activates PKC, which is responsible for the induction of NF-kB, hence the transcription of cytokines involved in T lymphocyte activity. Most immune cells express VDR and have the enzyme CYP27B1. The circulating levels of VD are too low to influence immune responses in vivo, and the immune upregulation by calcitriol is dose-dependent. Not only antigen-presenting cells but also activated T lymphocytes are able to produce 1,25-VD in concentrations high enough to influence the responsive genes of VD [43].

**Figure 3:**
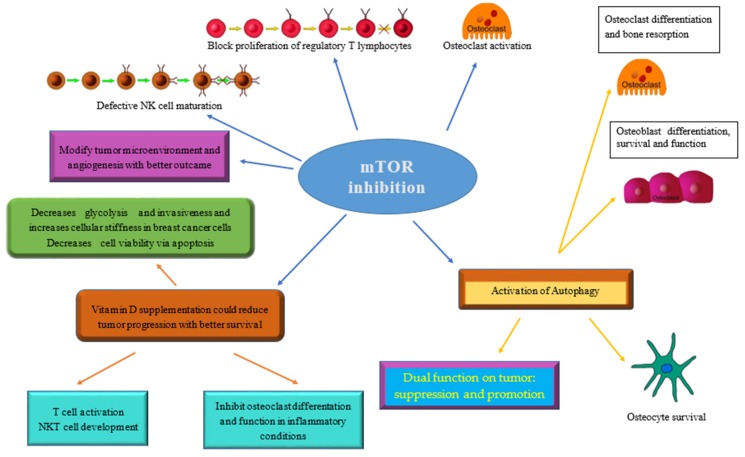
The main consequences of mTOR inhibition on tumor, bone, and the immune system are summarized along with how vitamin D supplementation can modulate its effects and contribute to achieving a better therapeutic goal.

It has already been reported that VDR signaling is required for T cell activation and NKT cell development, both of which are important immunological factors for antitumor responses [44,45]. More recently, it has also been demonstrated that VD decreases tumor growth and increases tumor infiltrating CD8+ T cells in breast cancer [46]. Figure 3 show the relationships between mTOR inhibition, cancer, the immune system, and bone.

## 6. Clinical Implications

Nowadays in oncology, mammalian target of rapamycin inhibitors (mTOR-I) represent widely used drugs, exerting their function by inhibiting a serine/threonine kinase with a pivotal role in cellular metabolism and in a wide range of eukaryotic biological/cellular functions and signaling networks. The major impact of mTOR inhibition on many biological functions essential to guarantee body homeostasis and survival explain its value in different therapeutic fields interconnected with each other, as are tumors, the immune system, and bone. From this perspective, it is easy to understand the therapeutic potential of these inhibitors, individually, combined with other molecules, and in different dosages and combinations capable of modulating their effect [47].

Although many molecules have been preclinically studied, only some inhibitors are currently approved for clinical treatment [48]. Depending on their mechanism and targets, mTOR inhibitors can be classified into first and second generations. The first generation uses allosteric mechanisms to block the mTOR pathway, while the second generation prevents kinase activity in both mTORC1 and -2 using their target ATP binding site. Examples of the first generation include rapamycin and its analogs, while the second generation includes AZD8055, Torin1, PP242, and PP30. Until recently, several inhibitors targeting mTOR had been studied for their efficacy in tumor therapy; among these, Torin 1 and Torin 2 are new generation mTOR inhibitors demonstrated to be effective in several cancer types [49,50,51,52,53,54]. Little information is available in the literature about their activity on immune cells; it seems that healthy CD4+ lymphocytes are not affected during treatment for acute T cell leukemia [55].

Rapamycin is the famous specific mTOR inhibitor, which inhibits mTOR complexes depending on the dose being used. Different doses of rapamycin are needed to suppress mTOR in different cell lines and the phosphorylation of different mTOR substrates, while mTORC1 and mTORC2 show different sensitivities to this molecule. mTORC2 is generally resistant to rapamycin even at micro-molar doses; however, prolonged treatment with nano-molar doses of rapamycin can lead to suppression of mTORC2 in several human cancer cell lines [56].

It has been shown that everolimus induces a reduction of CD4+ cells and an increase in the Treg population in a dose-dependent manner in metastatic prostate cancer patients [57]. Considering the heterogeneity and complexity of the immune system and, in our case, of the interactions between this system, bone, and tumor cells, although significant data are lacking in this regard, we consider it very likely that even on these cells or rather on these complex systems, different doses of rapamycin may have different effects. This could be a precious opportunity to achieve different specificities in different conditions and with different dosages.

**Figure 4:**
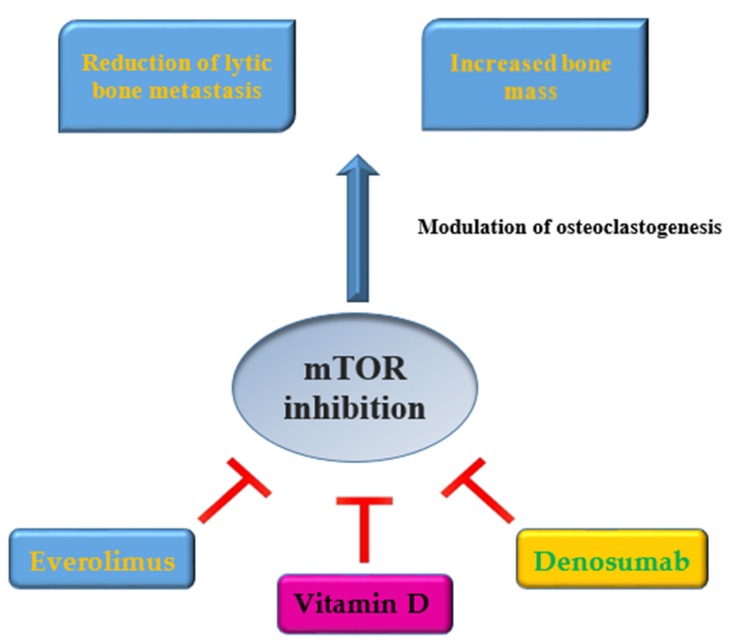
Inhibition of mTOR in cancer and bone effects: Everolimus, Vitamin D and Denosumab together contribute to inhibiting cancer progression and modulate osteoclastogenesis.

Everolimus has been approved as an oral mTOR inhibitor for advanced renal cell carcinoma, metastatic tumors of the pancreatic neuroendocrine and advanced hormone receptor (HR) positive/HER2 negative breast cancer. Side effects of everolimus include dermal toxicity, mucositis, hypertriglyceridemia, hypercholesterolemia, nausea, fatigue, anemia, and neutropenia. Furthermore, temsirolimus, indicated in advanced renal cell carcinoma, has an intense pulmonary toxicity. Other rare side effects include the risk of secondary lymphoma, interstitial lung disease, and the reactivation of latent infections [6]. In clinical practice, in a phase III study, BOLERO-2, the mTOR inhibitor everolimus demonstrated, at a median follow-up of 18 months, a longer median progression-free survival (primary endpoint) in a statistically significant way in combination with exemestane versus exemestane alone, in postmenopausal patients with HR positive/HER2 negative metastatic breast cancer, with disease progressed to therapy with non-steroidal aromatase inhibitors. In the arm of patients treated only with exemestane, increases in the levels of bone resorption and formation markers were recorded at weeks 6 and 12 compared to baseline. Meanwhile, in the combination arm, there was a decrease in these levels of bone markers, and the effects of everolimus were not influenced by the use of bisphosphonate [58]. The phase IIIb 4EVER study evaluated the combination everolimus/exemestane in postmenopausal patients with HR positive/HER2 negative metastatic breast cancer in terms of efficacy, safety, and quality of life in a large patient population, i.e., without limitations on the number of previous chemotherapy lines, the time point of progression after nonsteroidal aromatase inhibitors, and previous exemestane. This study also demonstrated a favorable impact of everolimus on bone turnover, versus exemestane, through the assessment of changes in bone turnover biomarkers [59]. In a retrospective analysis, immune checkpoint inhibitors combined with denosumab in patients with metastatic melanoma demonstrated a statistically significant advantage in terms of progression-free survival and overall survival [31]. An association between VD deficiency and worse outcomes in patients with metastatic melanoma but not in patients with non-metastatic melanoma has been demonstrated, suggesting an antigen-dependent immunological effect of VD. Furthermore, significantly worse outcomes were recorded in baseline VD-deficient patients (≤20 ng/mL) compared to patients with baseline VD levels in the normal ranges. The PROVIDENCE trial, a multicentric and prospective trial, is underway, which aims to investigate the role of hypovitaminosis D and the integration of VD on treatment outcome in patients with advanced cancer subjected to immunotherapy (primary endpoint: failure of treatment over time) [60]. Assessing 25-VD levels and the VDBP rs7041 genotype, before starting therapy, and quantifying nivolumab concentrations at 15 days, to eventually change the immunotherapeutic dosage or predict VD supplementation, could reduce the risk of progression of the tumor [38]. In squamous cell carcinoma of the head and neck, low VD levels have been associated with lymph node metastases and a negative HPV state; moreover, low levels of VD seem to predict poor overall survival [61]. Figure 4 synthesizes the effects of everolimus, VD, denosumab, and mTOR inhibition on bone.

## 7. Conclusions

The significant role of mTOR in driving several processes in tumorigenesis by controlling protein synthesis, growth, proliferation, and survival in cancer cells on the one hand, and by affecting the characterization and activity of the tumor microenvironment on the other hand, offers an important therapeutic perspective. Understanding mTOR activities in cancer is the basis to develop new antitumor therapy strategies. In particular, with a look through the actions of mTOR on the immune system and on bone metabolism, we focused on the possible modulation of osteclastogenesis by its inhibitors. Indeed, preclinical studies showed the ability of mTOR inhibition to reduce the number of lytic bone metastases and to increase bone mass in tumor-bearing mice. The inhibition of this molecule by multiple activities, obviously interferes with each of these and specifically in our case, we refer to the transduction process of RANK-RANKL and 1,25-VD. Inhibiting mTOR, we inhibit not only the survival, proliferation, senescence, and metabolism of the cancer cell but also the ability to activate the osteoclast. This goal could be achieved both by mTOR inhibitors such as everolimus, and with denosumab and VD. Furthermore, mTOR is a regulator of cytotoxic T lymphocyte differentiation and of natural killer cells as well as a modulator of the role of PD-L1. Combination studies of mTOR inhibitors and immunotherapy are necessary to confirm the validity of the biological rationale presented in this paper, in order to bring advantages in outcome for patients affected by solid tumors in the metastatic phase. Discussing the role of the mTOR pathway in the immune system, cancer, and bone metabolism could help to explore the mechanism of action of novel potential therapeutic agents for which further investigation is needed.

## Abbreviations

PI3K	phosphoinositide 3-kinase
AKT	protein kinase B
mTOR	mammalian target of rapamycin
TME	umor microenvironment
IL-10	Interleukin-10
TGF-β	transforming growth factor
CTLA-4	cytotoxic T-Lymphocyte protein 4
PD-1	Programmed Death 1
TOR1	target of rapamycin 1
mTORC1/2	mTOR complex ½
Raptor	regulatory-associated protein of mTOR
mLST8	mammalian lethal with sec-13 protein 8
DEPTOR	DEP domain-containing mTOR-interacting protein
S6K1	ribosomal protein S6 kinase B1
RPS6	ribosomal protein S6
PRAS40	Proline-rich-AKT-substrate 40kDa
Rictor	rapamycin-insensitive companion of mTOR
mSin1	mammalian stress-activated map kinase-interacting protein 1
Protor1/2	protein observed with Rictor 1 and 2
M-CSF	macrophage colony-stimulating factor
4E-BP1	4E-binding protein 1
TSC1	tuberic complex sclerosis complex-1
eIF4E	starting factor of eukaryotic translation 4E
CSLCs	cancer stem cells
ADCC	antibody-dependent cellular cytotoxicity
OPG	Osteoprotegerin
M-CSF	macrophage colony-stimulating factor
VD	Vitamin D
25-VD	25-hydroxy-vitamin D
VDBP	vitamin D-binding protein
Protor1/2	protein observed with Rictor 1 and 2
TSC1/2	tuberous sclerosis 1 and 2
AMPK	AMP-activated protein kinase
PTEN	phosphatase and tensin homologue
TSC	tuberous sclerosis complex
p-mTOR	phosphorylated mTOR
p-4EBP1	phosphorylated 4EBP1
eIF4E	eukaryotic translation initiation factor 4E
PIK3CA	phosphoinositide 3-kinase catalytic subunit alpha
PIK3CB	phosphoinositide 3-kinase catalytic subunit beta isoform
RPS6KA3	ribosomal protein S6 kinase A3
IGF1R	insulin-like growth factor 1 receptor
VEGF	vascular endothelial growth factor
p-Akt	phosphorylated Akt
S6K1	ribosomal protein S6 kinase B1
RPS6	ribosomal protein S6
ncRNAs	non-coding RNAs
lncRNA	long non-coding RNA
PIK3CD	phosphoinositide 3-kinase catalytic subunit delta
PIK3R3	phosphoinositide-3-kinase regulatory subunit 3
